# Transketolase promotes colorectal cancer metastasis through regulating AKT phosphorylation

**DOI:** 10.1038/s41419-022-04575-5

**Published:** 2022-02-02

**Authors:** Minle Li, Xue Zhao, Hongmei Yong, Jian Xu, Pengfei Qu, Shuxi Qiao, Pingfu Hou, Zhongwei Li, Sufang Chu, Junnian Zheng, Jin Bai

**Affiliations:** 1 Cancer Institute, Xuzhou Medical University, Xuzhou, Jiangsu China; 2 Department of Oncology, The Affiliated Huai’an Hospital of Xuzhou Medical University and The second People’s Hospital of Huai’an, Huai’an, 223002 China; 3 Center of Clinical Oncology, Affiliated Hospital of Xuzhou Medical University, Xuzhou, Jiangsu China

**Keywords:** Cancer, Cell biology

## Abstract

Transketolase (TKT) which is an important metabolic enzyme in the pentose phosphate pathway (PPP) participates in maintaining ribose 5-phosphate levels. TKT is necessary for maintaining cell growth. However, we found that in addition to this, TKT can also affect tumor progression through other ways. Our previous study indicate that TKT could promote the development of liver cancer by affecting bile acid metabolism. And in this study, we discovered that TKT expression was remarkably upregulated in colorectal cancer, abnormal high expression of TKT is associated with poor prognosis of colorectal cancer. Additionally, TKT promoted colorectal cancer cell growth and metastasis. Further study demonstrated that TKT interacted with GRP78 and promoted colorectal cancer cell glycolysis through increasing AKT phosphorylation, thereby enhancing colorectal cancer cell metastasis. Thus, TKT is expected to become an indicator for judging the prognosis of colorectal cancer, and provide a theoretical basis for drug development of new treatment targets for colorectal cancer.

## Introduction

One of the characteristics of malignant tumors is enhanced aerobic glycolysis [1]. Emerging studies have revealed that glycolysis is positively correlated with the malignant progression of cancer [2, 3]. It is evident that lactate promotes tumor growth and metastasis [4–6]. Thus, targeting glycolysis remains attractive for cancer therapy.

Colorectal cancer (CRC) is a malignant tumor of the digestive system [7, 8], and about half of patients with CRC die due to tumor metastasis [9]. Therefore, finding a potential therapeutic target for CRC metastasis is important for clinical treatment.

The transketolase (TKT) family includes three members of TKT, TKTL1, and TKTL2. In recent years, a lot of research is focused on TKTL1, and find that TKTL1 is highly expressed in lung cancer, cervical cancer, esophageal squamous cell carcinoma. [10–13]. TKTL1 is positively associated with tumor development and poor prognosis [14–16]. However, few studies have focused on TKT, actually, in most tumors, TKT is the highest-expressing member of the transketolase family [17]. In hepatocellular carcinoma (HCC), TKT knockdown causes R5P accumulation but inhibits cell proliferation, the explanation for this phenomenon is that the absence of TKT results in a decrease in NADPH, which disrupts the cell’s redox balance and causes an increase in ROS [18]. Some studies have pointed out that high expression of TKT in cervical cancer and pancreatic cancer enhance the PPP activity, thereby providing raw materials for the rapid growth and proliferation of tumor cells [19, 20]. In the above studies, TKT played its role through the PPP. However, in recent years, TKT’s functions other than promoting R5P generation have attracted people’s attention. For example, in breast cancer, TKT promotes metastasis through regulatingα-Ketoglutarate signaling pathway [21], and it has been shown that TKT enters the nucleus and activates the EGFR pathway, thereby promoting proliferation, viability, and migration of liver cancer [22]. Furthermore, our previous research also confirms that TKT transports into the nuclear to inhibit FXR promoter activity, affects liver bile acid metabolism, and promotes liver cancer [23]. Nevertheless, the function of TKT and related mechanisms in CRC are currently unclear.

In the present study, we used tissue microarrays (TMAs) of CRC patients with prognostic information to explore the function of TKT in CRC. We evaluated that TKT could be used as an independent prognostic indicator for CRC treatment, and we confirmed that TKT promoted CRC cells growth and metastasis. The function and related molecular mechanism of TKT in CRC metastasis were firstly elucidated in this study.

## Results

### TKT was highly expressed in CRC and cell lines and indicated poor prognosis

**Fig. 1:**
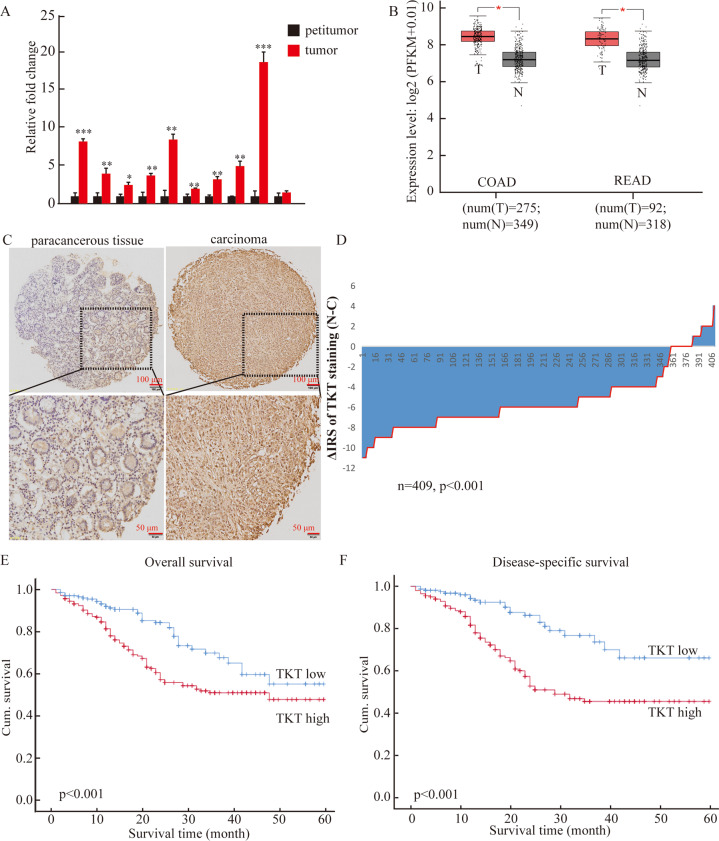
TKT is elevated in CRC, and is related to poor OS and DFS in CRC. (**A**) Detection of TKT expression in 10 pairs of colorectal cancer CRC and adjacent tissues. (**B**) In the Gene Expression Profiling Interactive Analysis (GEPIA), TKT expression was high in CRC. (**C**) TKT immunostaining in TMAs. Note: magnification ×100. (**D**) Distribution of TKT staining intensity difference in tumor and tumor-adjacent tissues. Note: N, tumor-adjacent tissues; C, tumor tissues, P < 0.001. (**E**) High TKT expression is regulated to poorer overall survival of patients with CRC (*P* < 0.001). (**F**) High TKT expression is regulated to poorer disease-specific survival of colorectal carcinoma patients (*P* < 0.001, log-rank test).

We tested TKT expression in 10 pairs of colorectal cancer and adjacent samples. The data demonstrated that compared with adjacent normal tissues, TKT expression was significantly upregulated in tumor tissues, and increased TKT expression was also detected in the Gene Expression Profiling Interactive Analysis (GEPIA) (Fig. 1A, B). Consistently, compared with FHC (normal colorectal epithelial cell), TKT expression was higher in CRC cell lines (HT-29, DLD1, LOVO, SW620, HCT116, SW480) (Fig. S1A).

To clarify the relationship between TKT and the prognosis of CRC, we used immunohistochemical (IHC) analysis to evaluate the level of TKT expression in 464 (81.7%) of 568 CRC samples and 401 (70.6%) of 568 nontumor tissues. The expression of TKT in tumor tissues of patients with CRC is significantly higher than adjacent tissues (Fig. 1C). Next, we analyzed the paired cancer and para-cancerous tissues of 409 patients, and the data suggested that TKT expression was significantly increased in cancerous tissue (Fig. 1D).

To further clarify the significance of TKT in the clinical treatment of CRC, we used Fisher’s exact test to detect the correlation between TKT expression and clinicopathological characteristics of CRC. Immunoreactivity score (IRS)1–6 were classified as TKT low expression, and IRS 8–12 were classified as TKT high expression. The data showed that TKT was low in 45% (209/464) and high in 55% (255/464) of CRC tissues. The obvious positive correlation with the high expression of TKT were tumor lymph node metastasis (*P* < 0.001) and tumor node metastasis (TNM) stage (*P* < 0.001). However, TKT expression had no correlation with age, gender, depth of invasion, tumor diameter, or differentiation (Table 1).

**Table 1:** Relationship between TKT expression and clinicopathological features of CRC patients.

Variables	TKT expression (*n* = 464 cases)		
	Low (%)	High (%)	*P*^a^
All patients	209 (45)	255 (55)	
Age (years)			0.454
<63	105 (43)	137 (57)	
≥63	104 (47)	118 (53)	
Gender			0.878
Males	119 (45)	147 (55)	
Females	90 (45)	108 (55)	
Depth of invasion			0.695
T1/T2	134 (46)	159 (54)	
T3/T4	75 (44)	96 (56)	
Lymph node metastasis			<0.001
N0	158 (54)	137 (46)	
N1/N2/N3	51 (30)	118 (70)	
Distant metastasis			0.360
M0	198 (45)	246 (55)	
M1	11 (55)	9 (45)	
TNM stage			<0.001
I/II	158 (56)	125 (44)	
III/ IV	51 (28)	130 (72)	
Tumor diameter		0.982	
<5 cm	151 (45)	184 (55)	
≥5 cm	58 (45)	71 (55)	
Differentiation			0.658
Poor	30 (48)	33 (52)	
Moderate/high	179 (45)	222 (55)	

Kaplan–Meier survival analysis demonstrated that patients with higher TKT expression had poorer overall survival (Fig. 1E). Moreover, we used the KM-plotter to do bioinformatics analysis, the result also showed that CRC patients with higher TKT expression had shorter disease-specific survival (Fig. 1F).

To confirm whether TKT can be an independent indicator for judging the prognosis of CRC, Univariate Cox regression was used to analyze the value of TKT in the prognostic evaluation of CRC. The result showed that TKT expression, lymph node metastasis (LNM), and TNM stage, were the significant prognostic indicators for the OS of CRC patients. Moreover, we used univariate Cox regression analysis to find that in the disease-specific survival (DFS) of CRC patients, TKT expression, LNM, and TNM stage were the significant prognostic indicators in the disease-specific survival (DFS) of CRC patients (Table 2). Our data confirmed that TKT high expression may serve as a potential independent prognostic indicator in CRC.

**Table 2:** Univariate Cox regression analysis of TKT expression and clinicopathologic variables predicting the survival of CRC patients.

Variable*	Overall survival			Disease-specific survival		
	Hazard ratio	95% CI^†^	P*	Hazard ratio	95% CI^†^	P*
TKT	1.981	1.365–2.875	<0.001	1.881	1.277–2.771	0.001
Age	1.454	1.035–2.041	0.031	1.505	1.020–2.221	0.040
Gender	1.441	1.028–2.021	0.034	1.476	1.003–2.173	0.048
LNM	1.756	1.253–2.462	0.001	1.970	1.336–2.904	0.001
TNM stage	1.880	1.341–2.635	<0.001	1.881	1.277–2.771	0.001
Differentiate	0.534	0.307–0.931	0.027	0.616	0.329–1.151	0.129
Tumor diameter	1.114	0.768–1.617	0.570	0.985	0.635–1.528	0.945
Depth of invasion	1.523	1.081–2.147	0.016	1.449	0.979–2.146	0.064

### TKT promoted CRC cells proliferation

**Fig. 2:**
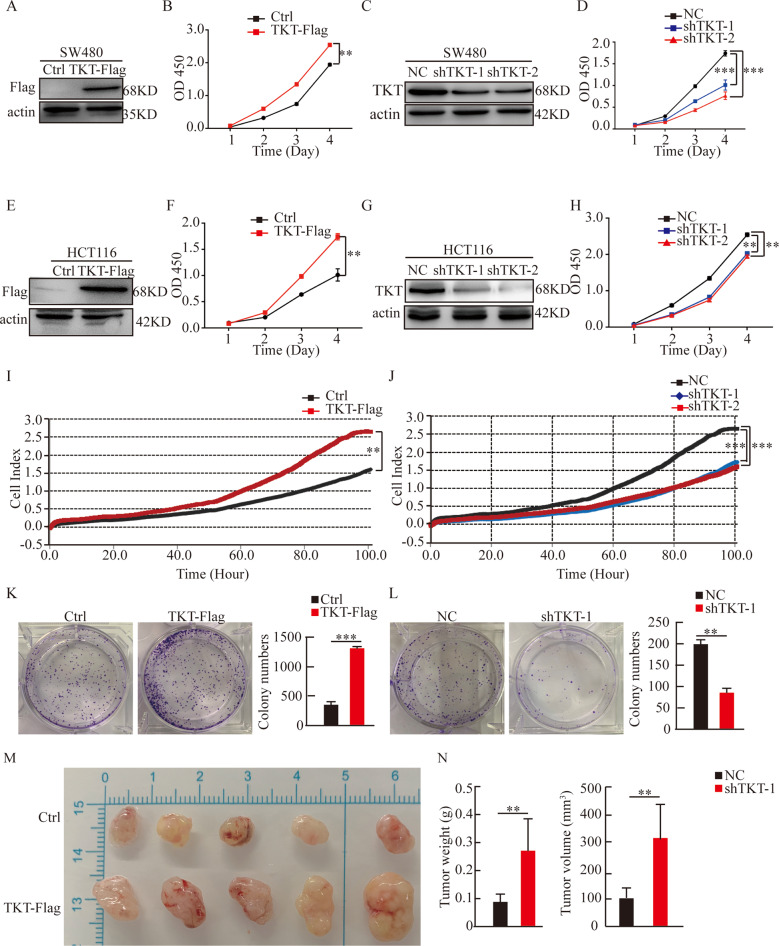
TKT promotes CRC cell proliferation. (**A**) Overexpression of TKT was verified in SW480. (**B**) TKT overexpression enhanced SW480 proliferation ability. (**C**) Knockdown of TKT was confirmed in SW480. (**D**) TKT knockdown significantly reduced SW480 proliferation. (**E**) Overexpression of TKT was confirmed in HCT116. (**F**) TKT overexpression enhanced HCT116 proliferation ability. (**G**) Knockdown of TKT was confirmed in HCT116. (**H**) TKT knockdown significantly reduced HCT116 proliferation. (**I**) RTCA indicated that TKT overexpression enhanced HCT116 proliferation ability. (**J**) RTCA indicated that TKT knockdown inhibited HCT116 proliferation. (**K**–**L**) Colony formation assays. *P < 0.05, **P < 0.01, ***P < 0.001. (**M**–**N**) Evaluated the role of TKT in tumor formation by measuring tumor volume and weight (**p < 0.01).

Because previous results indicated that TKT affected the malignancy of colorectal cancer, next, we wanted to explore the function of TKT in CRC. We established SW480 and HCT116 stable cell lines with TKT overexpression or knockdown. Then, we used the cell counting kit-8 assays (CCK-8) to examine the effect of TKT on proliferation. The result suggested that overexpression of TKT significantly promoted cell proliferation. Conversely, knockdown of TKT alleviated cell proliferation (Fig. 2A–H). Moreover, we used RTCA and colony formation assays to further confirm that TKT promoted colorectal cancer cells proliferation (Fig. 2I–L). Furthermore, the xenograft experiments showed that the tumor volume of TKT overexpression group was notably bigger than the control group (Fig. 2M, N). In summary, these results demonstrated that TKT stimulated the growth of CRC cells.

### TKT promoted metastasis of CRC cells

**Fig. 3:**
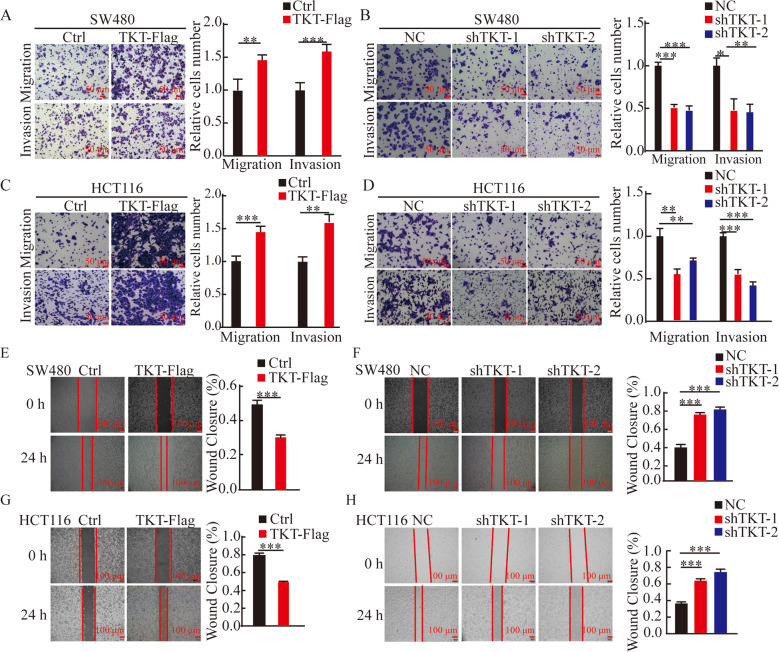
TKT promoted metastasis of CRC cells. (**A**–**D**) Migration and invasion detection of SW480 and HCT116 cells with TKT overexpression and knockdown. (**E**–**H**) Wound-healing assays. *P < 0.05, **P < 0.01, ***P < 0.001.

Then, we investigated whether TKT affects the migration and invasion ability of CRC cells. The transwell assays dispalyed that TKT overexpression significantly enhanced migration and invasion ability (Fig. 3A, C), the wound-healing assay also confirmed this phenomenon (Fig. 3E, G). In contrast, the migration and invasion ability of cells weakened with the decrease of TKT expression (Fig. 3B, D, F, H). Furthermore, we observed the same phenomenon in HCT116 via RTCA assay (Fig. S2A-D).

**Fig. 4:**
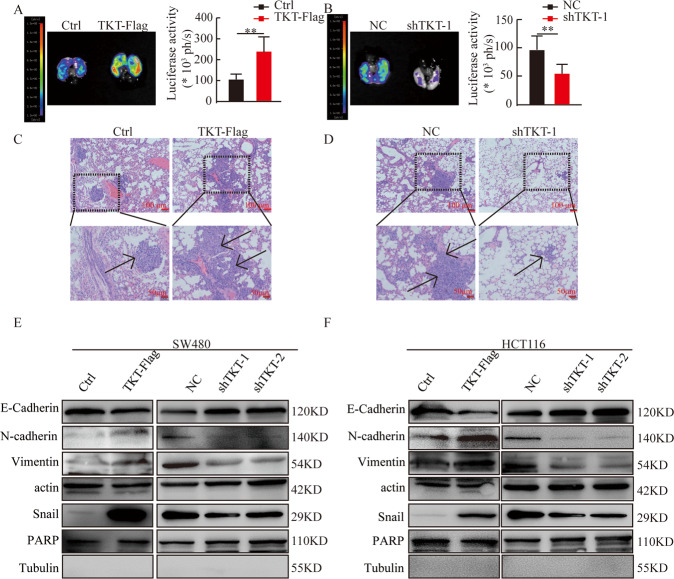
TKT promotes CRC cell metastasis. (**A** and **B**) Representative pictures and analysis of lung metastases. *P < 0.05, **P < 0.01, ***P < 0.001. (**C** and **D**) Corresponding H&E-stained lung. The arrow pointed to the metastasis. (**E** and **F**) Protein levels of EMT markers in SW480 and HCT116. *P < 0.05, **P < 0.01, ***P < 0.001.

We used tail vein injection of lung metastasis model to test the role of TKT in colorectal cell metastasis in vivo. 1 × 10^6^ HCT116 stable cells with TKT overexpression or knockdown were injected to 8-week-old nude mice, and the lung metastases was detected after 2 months. Compared with the control group, the fluorescence intensity of the TKT overexpression group was stronger and more metastatic foci formed in the lungs (Fig. 4A, C), but TKT knockdown significantly reduced the fluorescence intensity and the number of metastatic foci (Fig. 4B, D).

Angiogenesis plays an important role in tumor growth and metastasis [24]. Because our data suggested that high TKT expression was an essential factor for cancer metastasis, we studied the role of TKT in CRC angiogenesis. We used HUVECs to do blood vessel formation experiments. And we found that the number of complete tubular structures was increased in the medium collected from TKT overexpression cells and reduced in the medium collected from TKT knockdown cells compared with the corresponding controls (Fig. S3A–D).

Epithelial mesenchymal-transition (EMT) can reduce cell-cell adhesion, promote cell migration and invasion, thereby promoting tumor metastasis [25]. We found that TKT overexpression promoted EMT markers (N-cadherin, Vimentin, and Snail) expression but suppressed the level of E-cadherin. By contrast, TKT knockdown upregulated E-cadherin expression and inhibited N-cadherin, Vimentin, and Snail expression (Fig. 4E, F). These data strongly proved that TKT promoted CRC migration, invasion, and metastasis.

### TKT promoted CRC cells metastasis through activating aerobic glycolysis

**Fig. 5:**
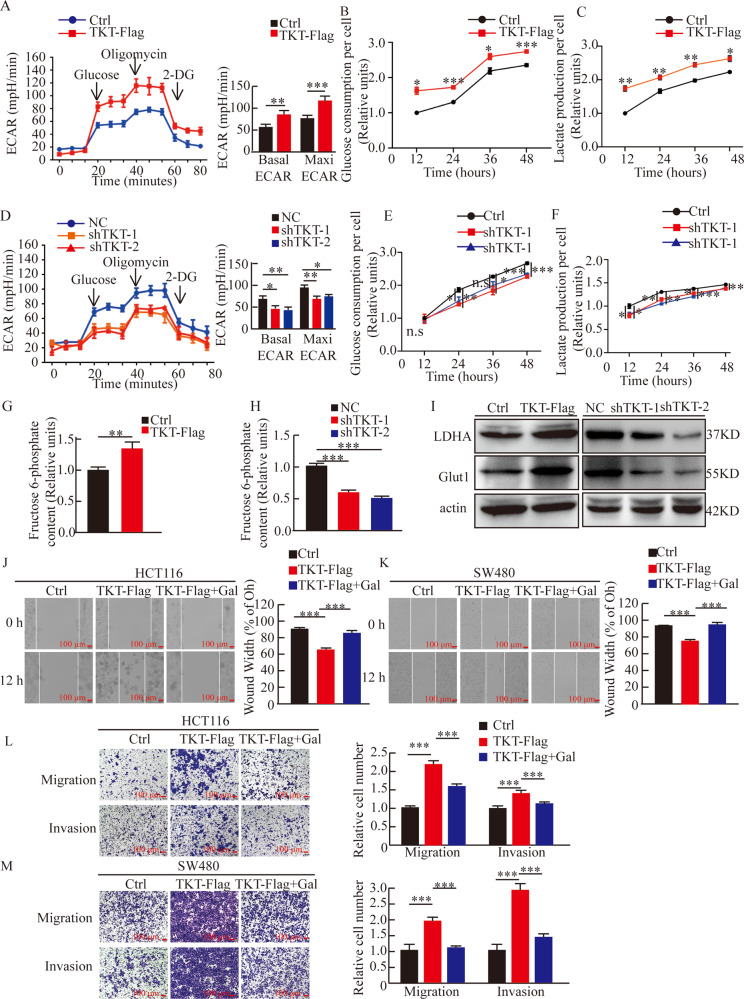
TKT promoted CRC cells metastasis through activating aerobic glycolysis. (**A**) Seahorse assays showed that TKT overexpression significantly promoted aerobic glycolysis. Left, representative curve, Right, quantification of three independent experiment of basal ECAR and maxi ECAR. (**B**–**C**) TKT promoted glucose consumption and lactate production in HCT116. (**D**) Seahorse assays showed that TKT knockdown significantly inhibited aerobic glycolysis, Left, representative curve, Right, quantification of three independent experiment of basal ECAR and maxi ECAR. (**E**–**F**) Relative glucose consumption and lactate production were detected in HCT116. (**G**–**H**) The content of F6P was detected in HCT116. (**I**) TKT promoted the expression of Glut1 and LDHA. (**J**–**M**) Wound-healing, migration and matrigel invasion assays were performed in HCT116 and SW480, *P < 0.05, **P < 0.01, ***P < 0.001.

Tumor metastasis is usually accompanied by high-intensity glycolysis. Our results showed that TKT significantly increased aerobic glycolysis, glucose consumption, lactic acid production of CRC cells as expected (Fig. 5A–C). Conversely, TKT knockdown reduced aerobic glycolysis, glucose consumption, lactic acid (Fig. 5D–F). In addition, we found that TKT overexpression also resulted in increased fructose 6-phosphate (F6P) production, which is the intermediate of the non-oxidative phase (Fig. 5G, H). It is well known that F6P can enter the glycolytic pathway and then be metabolized, this result further confirmed that TKT overexpression could promote glycolysis. Moreover, we detected the expression of genes regulating glucose metabolism. The data showed that TKT overexpression enhanced Glut1 and LDHA expression while knockdown of TKT inhibited these genes (Fig. 5I).

In order to test whether TKT’s promotion of colorectal cancer metastasis is dependent on glycolysis, we used galactose which induced cells to survive on ATP provided by mitochondrial respiration instead of glucose. We found that decreased glycolysis caused by galactose significantly attenuated migration and invasion promoted by TKT overexpression (Fig. 5J–M). And the increased expression of EMT marker induced by TKT overexpression was also reversed (Fig. S4A). These results implied that TKT may exert its function of promoting metastasis in CRC cells through activating aerobic glycolysis.

### TKT promoted aerobic glycolysis of CRC cells by promoting AKT phosphorylation

To investigate the possible mechanism of TKT in CRC metastasis, we determined the possible genes from the previous mass spectrometry results [23] and focused on GRP78 because GRP78 promotes tumor growth and migration via regulating AKT phosphorylation [26]. We suspected that TKT regulated AKT phosphorylation through interacting with GRP78 and then promoted aerobic glycolysis, thereby facilitated CRC cell metastasis.

**Fig. 6:**
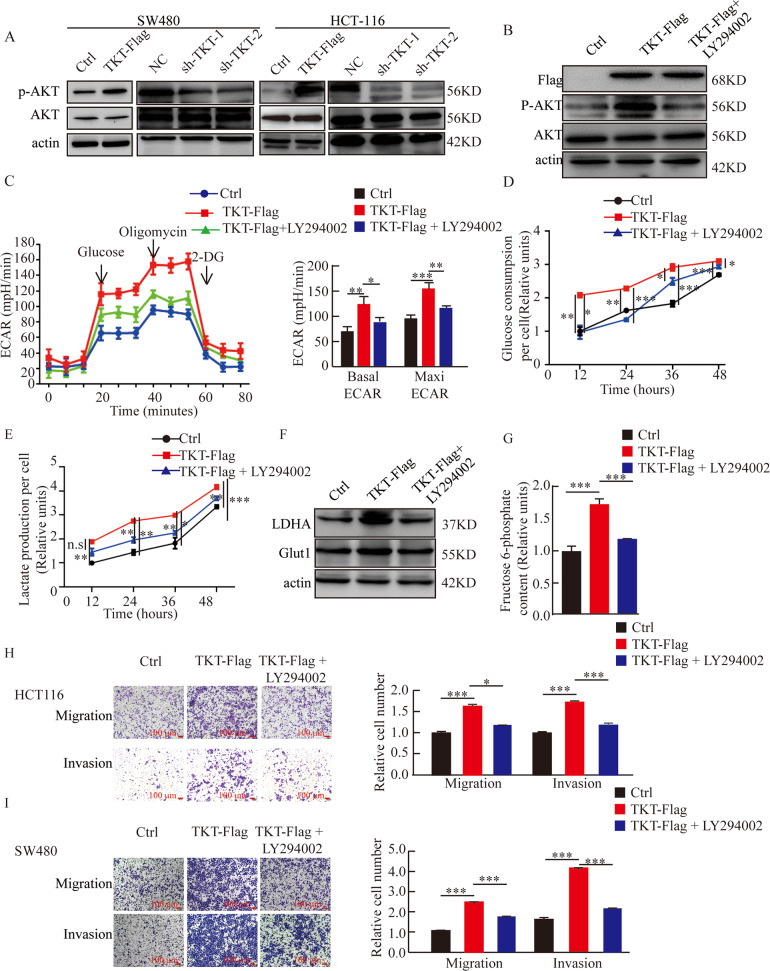
TKT promotes aerobic glycolysis of CRC cells by promoting AKT phosphorylation. (**A**) Expressions of AKT phosphorylation in SW480 and HCT116 cells with TKT OE or KD. (**B**) LY294002 effectively inhibited AKT phosphorylation in HCT116. (**C**) The regulation of glycolysis by TKT is dependent on AKT phosphorylation, Left, representative curve, Right, quantification of three independent experiment of basal ECAR and maxi ECAR. (**D**–**E**) Relative glucose consumption and lactate production were detected in HCT116. (**F**) Detecting the protein levels of LDHA and Glut1 by using Western blot. (**G**) The content of F6P was detected in HCT116. (**H**–**I**) Migration and invasion detection with AKT phosphorylation inhibitor (LY294002). *P < 0.05, **P < 0.01, ***P < 0.001.

Next, we evaluated the effect of TKT on AKT phosphorylation. The results showed that in SW480 and HCT116, the overexpression of TKT promoted AKT phosphorylation, and TKT reduction decreased AKT phosphorylation (Fig. 6A). To determine whether AKT phosphorylation has an effect on cell metabolism, we used drugs (LY294002) to inhibit AKT phosphorylation (Fig. 6B). We found that when AKT phosphorylation was inhibited, the enhanced aerobic glycolysis induced by elevated TKT expression was reversed (Fig. 6C–G). In addition, we showed that inhibition of AKT phosphorylation significantly reduced cell migration and invasion ability induced by TKT high expression (Fig. 6H, I). These results indicated that the regulation of TKT on glycolysis was dependent on AKT phosphorylation,

### Regulation of TKT to AKT phosphorylation and aerobic glycolysis was dependent on GRP78

**Fig. 7:**
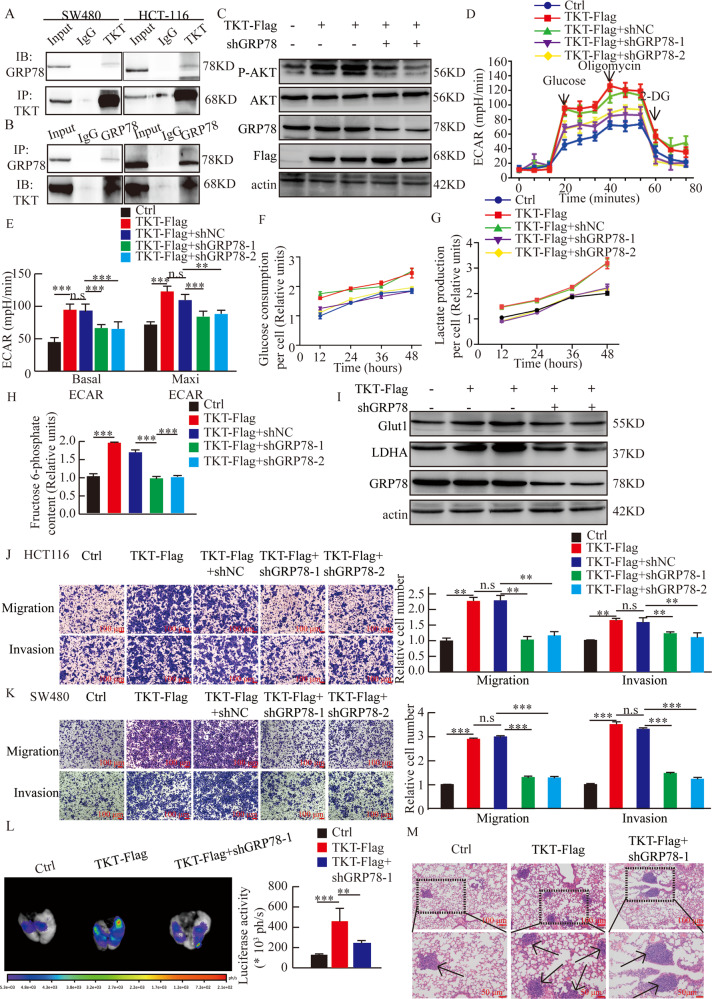
Regulation of TKT to AKT phosphorylation and aerobic glycolysis is dependent on GRP78. (**A**–**B**) Endogenous TKT coimmunoprecipitates with GRP78 in SW480 and HCT116. (**C**) The regulation of TKT to AKT phosphorylation was dependent on GRP78. (**D**–**E**) Regulation of TKT to aerobic glycolysis is dependent on GRP78. (**F**) Relative glucose consumption was determined in HCT116 cells. 12 h: Ctrl vs. TKT-Flag: *; TKT-Flag vs TKT-Flag +NC: n.s; TKT + NC vs TKT + shGRP78-1: **, TKT + NC vs TKT + shGRP78-2: **. 24 h: Ctrl vs. TKT-Flag: **; TKT-Flag vs TKT-Flag +NC: n.s; TKT + NC vs TKT + shGRP78-1: *, TKT + NC vs TKT + shGRP78-2: *. 36 h: Ctrl vs. TKT-Flag: *; TKT-Flag vs TKT-Flag +NC: n.s; TKT + NC vs TKT + shGRP78-1: *, TKT + NC vs TKT + shGRP78-2: *. 48 h: Ctrl vs. TKT-Flag: *; TKT-Flag vs TKT-Flag +NC: n.s; TKT + NC vs TKT + shGRP78-1: **, TKT + NC vs TKT + shGRP78-2: *. (**G**) Relative lactate productionwas determined in HCT116 cells. 12 h: Ctrl vs. TKT-Flag: *; TKT-Flag vs TKT-Flag +NC: n.s; TKT + NC vs TKT + shGRP78-1: **, TKT + NC vs TKT + shGRP78-2: *.24 h: Ctrl vs. TKT-Flag: **; TKT-Flag vs TKT-Flag +NC: n.s; TKT + NC vs TKT + shGRP78-1: *, TKT + NC vs TKT + shGRP78-2: *. 36 h: Ctrl vs. TKT-Flag: **; TKT-Flag vs TKT-Flag +NC: n.s; TKT + NC vs TKT + shGRP78-1: **, TKT + NC vs TKT + shGRP78-2: **. 48 h: Ctrl vs. TKT-Flag: *; TKT-Flag vs TKT-Flag +NC: n.s; TKT + NC vs TKT + shGRP78-1: **, TKT + NC vs TKT + shGRP78-2: *. (**H**) The content of F6P was detected in HCT116. (**I**) Western blot analysis about the expression of Glut1 and LDHA. (**J**–**K**) Migration and invasion detection with TKT overexpression and GRP78 knockdown. (**L**) Representative pictures and analysis of lung metastases in mice. (**M**) Corresponding H&E -stained lung sections. Arrows denote lung metastasis. *P < 0.05, **P < 0.01, ***P < 0.001.

On the basis of the above results, we next determined whether GRP78 participated in the regulation of AKT phosphorylation by TKT. We verified the interaction between GRP78 and TKT by using coimmunoprecipitation in SW480 and HCT116 cells (Fig. 7A, B). Then, we found that the overexpression of TKT and knockdown of GRP78 resulted in decreased AKT phosphorylation (Fig. 7C). Moreover, with the knockdown of GRP78, the phenomenon of enhanced glycolysis, glucose consumption, F6P, and lactic acid production, as well as the expression of Glut1 and LDHA caused by the overexpression of TKT was also reversed (Fig. 7D-I).

To validate whether the interaction of TKT and GRP78 could result in functional consequences, we investigated the role of GRP78 in CRC migration and invasion. The results demonstrated that overexpression of TKT and knockdown of GRP78 lead to weakened migration and invasion ability (Fig. 7J–M). These data suggested that TKT regulated the metastasis of CRC cells by binding to GRP78, which affected AKT phosphorylation, which in turn affects cell glycolysis (Fig. S4B).

## Discussion

In the traditional concept, the role of TKT is mainly to participate in PPP and *de novo* nucleotide biosynthesis. As an important member of the PPP, TKT has been found highly expressed and plays an important role in tumor development. In our previous studies, we found that the incidence of liver cancer was decreased in liver-specific TKT knockout mice compared with control littermates. This observation was explained by TKT absence in hepatocytes protected the liver from DNA damage which is induced by diethylnitrosamine (DEN) [27]. Additionally, the metabolomic analysis suggested that loss of TKT caused a decreased bile acid. Moreover, we confirmed that TKT entered the nucleus by interacting with STAT1, then inhibited FXR expression via enhancing the interaction between HDAC3 and FXR promoter [23]. However, the efficacy of TKT in colorectal cancer has not yet been reported.

In most of the existing studies, TKT mainly plays its role through directly generating ribose 5-phosphate, providing raw materials for the rapid proliferation of cells. We do not deny that TKT can work in the metastasis of colorectal cancer through the PPP, but our focus is whether TKT can regulate colorectal cancer metastasis in other ways besides PPP. We found for the first time that the expression of TKT in CRC was significantly higher than that in adjacent tissues. Moreover, we demonstrated that high expression of TKT expression was positively correlated with the depth of invasion, LNM, distant metastasis, TNM stage, OS, and DFS. Additionally, the data also confirmed that high TKT expression was an independent indicator for CRC prognostic. These data demonstrate that TKT plays a vital role in colorectal cancer metastasis.

Metastasis is an important cause of death in CRC patients [28]. Previous studies have confirmed that TKT is related to tumor cell growth and metastasis [18, 19, 21]. We found that TKT promoted the proliferation of CRC cells and increased CRC migration and invasion abilities. We also demonstrated that TKT promoted the lung metastasis of CRC cells in vivo. These data clarified that TKT plays an important role in CRC development.

In addition, the present study elucidated how TKT affects the development of colorectal cancer. GRP78 plays an important role in the proliferation, invasion, and metastasis of cancer cells such as prostate, endometrial, and CRC [26, 29–32]. The relationship between GRP78 and AKT has been proven [33], AKT phosphorylation can be regulated by GRP78 [34]. Moreover, AKT activation was closely related to tumor glycolysis and metastasis [35]. In this work, our data demonstrated that TKT promoted AKT phosphorylation. However, this regulatory effect disappeared with the knockdown of GRP78. We also revealed that blocking GRP78 could significantly reduce TKT-induced CRC cell migration and invasion, which was consistent with the result using the AKT inhibitor LY294002. These data suggested that reducing the expression of TKT could effectively inhibit CRC metastasis.

## Materials and Methods

### Patients and samples collection

We collected the TMAs (568 pairs of colorectal cancer and adjacent tissues) from the Affiliated Hospital of Xuzhou Medical University. All the patients underwent radical surgery from April 2010 to March 2015. Clinical patient information was provided by the Affiliated Hospital of Xuzhou Medical University.

### Cell culture and treatment

The colorectal cancer cell lines was purchased from the Chinese Academy of Sciences Cell Bank. SW480 and HCT116 were cultured in 1640/DMEM medium. All medium are supplemented with 10% fetal bovine serum, 1% penicillin (100 U/ml), and 1% streptomycin (100 μg/ml), the culture instrument is a 37 °C incubator containing 5% CO_2_.

### Antibodies

The western blot method was indicated in the reference [36]. The antibodies against TKT, GRP78, Actin were purchased from Proteintech (11039–1-AP, 11587-1-AP, 20536-1-AP). E-cadherin, N-cadherin were purchased from BD Biosciences (610181, 610920). Snail (A11794, Abclonal), Glut1 (73015, CST), LDHA (3582, CST).

### Immunohistochemistry (IHC)

The IHC method is shown as previously reported [37]. The dilution ratio of TKT antibody is 1:100.

### IHC scoring method

Two pathologists scored TMA separately and all discrepancies that were resolved after discussion. TKT staining score was determined by combining the rate of positive cells and depending on the IRS. TKT immunostaining intensity score was classified as: negative: 0–3, weak: 1, moderate: 2, strong: 3); the percentage of positive cells was graded as 1 (0–25%), 2 (26–50%), 3 (51–75%), and 4 (76–100%). According to IRS, TKT expression levels were classified as follows:low: 0–6, high: 8–12.

### Real-time cellular analysis (RTCA)

The RTCA experiment was carried out according to the manufacturer’s instructions (ACEA Biosciences). 0.5 × 10^4^ cells/200 ml medium were seeded in each well, and monitored every 15 min. Experiments about proliferation and migration were done for 96 h and 36 h, invasion assays were done for 48 h.

### Cell migration, invasion, and wound-healing assays

Cell migration and invasion assays were performed as previously described [38]. For the migration assay, placed the transwell chambers (8.0 μm, Corning, NY, USA.) in a 24-well plate with complete medium, then 2 × 10^5^ cells/100 μl serum-free median planted to chambers, and incubated for 48 h. For the invasion assay, added matrigel (BD Biosciences) to chambers, then 4 × 10^5^ cells were seeded into chambers after 4 h, and incubated for 48 h.

We seeded cells in 6-well plates and cultivated until the density reaches 90% confluence. Then, used a sterile 10 μl pipette tip to scrathed each well, washed away floating cells, and then added medium with 1% FBS and photographed at 0 and 24 h.

### Stable cell line generation

We constructed the pCDH-CMV-MCS-EF1-GreenPuro-CD513B-TKT lentivirus plasmid, and purchased TKT and GRP78 short hairpin RNA (shRNA) (GenePharma). HCT116 and SW480 stable cell lines were obtained through lentivirus infection and puromysin screening.

The shRNAs against human TKT and GRP78 were purchased from GenePharma Technology (Shanghai, China). shRNAs were listed in below:

shTKT-1:

GCCGCCAAUACAAAGGGUATTUACCCUUUGUAUUGGCGGCTT

shTKT-2:

CCGGCAAAUACUUCGACAATTUUGUCGAAGUAUUUGCCGGTT

shGRP78-1:

GAGGUGUCAUGACCAAACUTTAGUUUGGUCAUGACACCUCTT

shGRP78-2:

GGGCAAAGAUGUCAGGAAATTUUUCCUGACAUCUUUGCCCTT

### Proliferation and colony formation assays

We used CCK-8 experiments to detect cell proliferation ability according to manufacturer’s protocol (Dojindo, Japan). Colony formation assay was performed in six-well plates, one thousand cells were seeded with complete medium, and stained with 0.1% crystal violet after 14 days, then counted the colony number.

### Seahorse assays, glucose uptake, F6P, and lactate production

We used Seahorse Bioscience Extracellular Flux Analyzer to detect glycosis via measuring extracellular acidification rate (ECAR) in real time. Basal ECAR is indicated by ECAR in the presence of glucose. Maxi ECAR is indicated by ECAR in the presence of Oligomycin.

Cells were plated in six-well plates. Culture medium was collected at 12 h, 24 h, 36 h, and 48 h. We used glucose and lactate assay kit (Shanghai Rongsheng Biotech, Shanghai, China) to measure the content of glucose and lactate following manufacturer’s instructions.

The content of F6P was measured by using the fructose-6-phosphate assay kit (MAK020, Sigma, USA).

### Animal work

BALB/c nude mice (Male, 8 weeks old) were purchased from Jicui Laboratory Animal Technology (Nanjing, China). All animal experiments were approved by the Experimental Animal Center of Xuzhou Medical University. Injected HCT116-Luc-shNC, HCT116-Luc-shTKT-1, HCT116-Luc-Ctrl, and HCT116-Luc-TKT-Flag cells (5 × 10^6^) into mice through the tail vein, and the fluorescence intensity in the mice was measured by live imaging after 8 weeks.

We injected HCT116-Ctrl, HCT116-TKT-Flag cells (2 ×10^6^) into mice subcutaneously, then measured the length (L) and width (W) of the tumor and calculated with the following formula: Tumor volume = (L × W^2^)/2 after 6 weeks.

### Statistical analysis

Statistical analysis were conducted using GraphPad Prism 7.0. Single-factor analysis for three groups were calculated using one-way ANOVA. Analysis for two groups were analyzed by two-tailed Student’s *t*-test. Data were presented as mean ± SEM, and the bar indicated the mean. *P* < 0.05 is statistically significant.

## Supplementary information

Supplementary figure 1

Supplementary figure 2

Supplementary figure 3

Supplementary figure 4

Supplementary figure legends

Aj-checklist

## Data Availability

The datasets are available from the corresponding author on reasonable request.
