# Supplementary figures and images for: Transketolase promotes colorectal cancer metastasis through regulating AKT phosphorylation
Source: Cell Death Dis. 2022 Feb 2;13(2):99. doi: 10.1038/s41419-022-04575-5 (PMC8810869; doi:10.1038/s41419-022-04575-5)

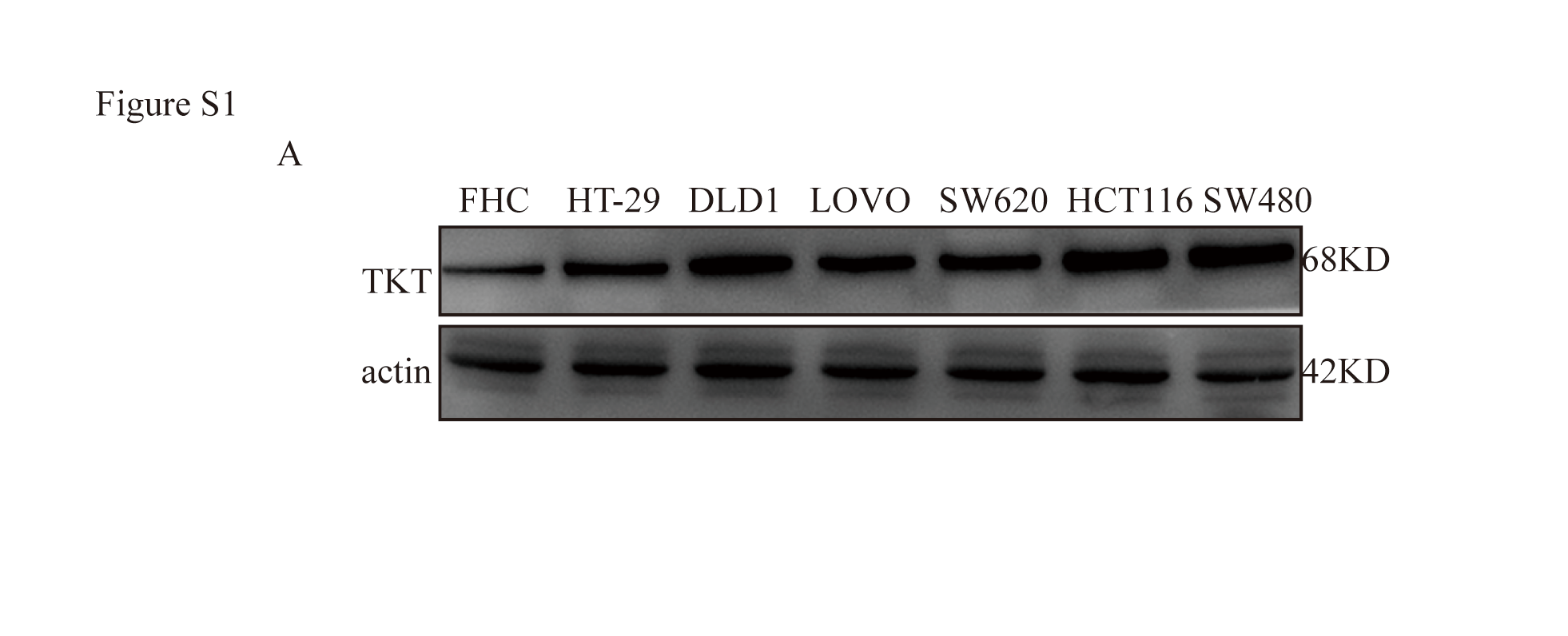

Supplement: Supplementary file 1 — Supplementary figure 1 [file 41419_2022_4575_MOESM1_ESM.png]

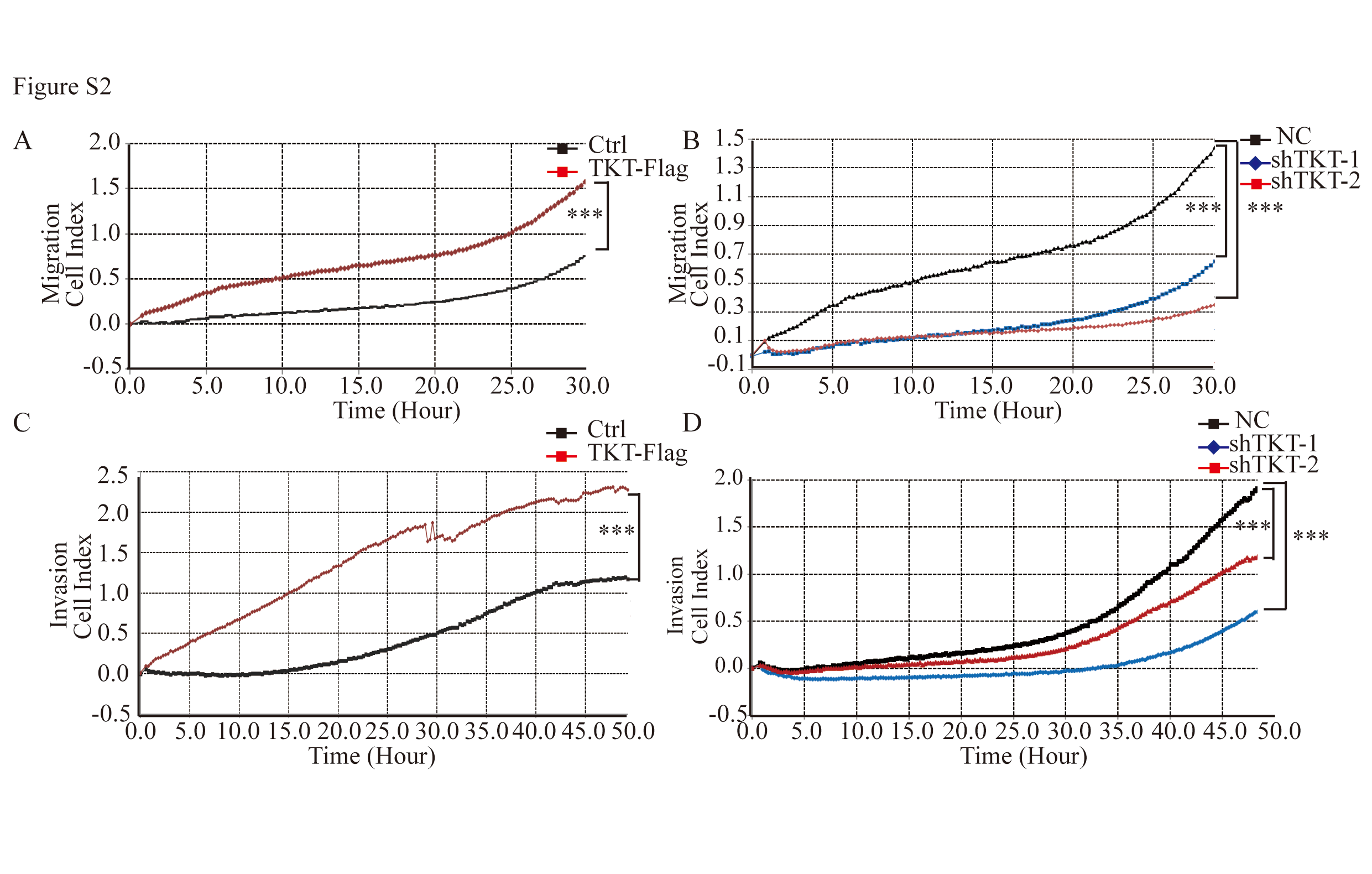

Supplement: Supplementary file 2 — Supplementary figure 2 [file 41419_2022_4575_MOESM2_ESM.png]

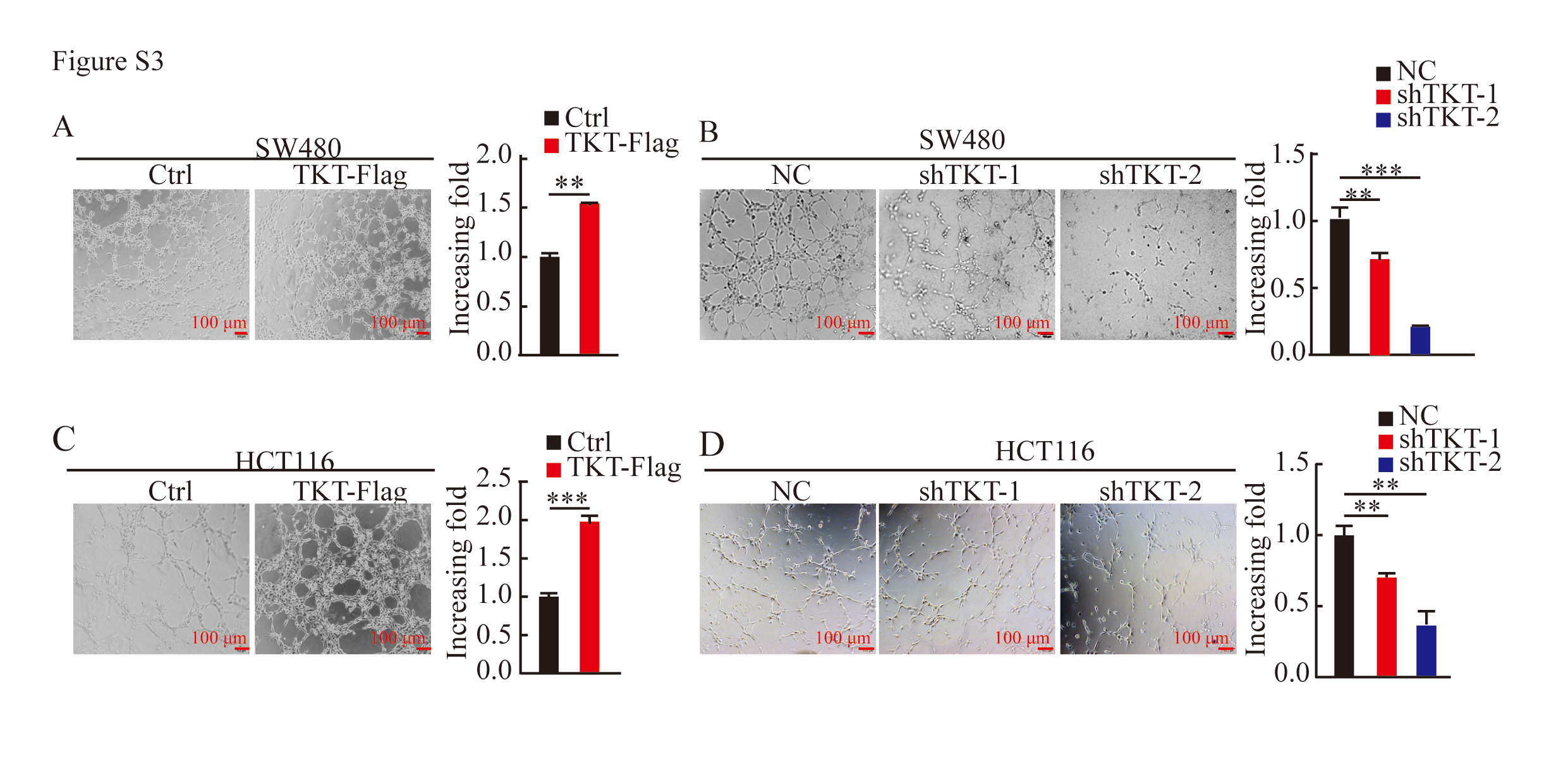

Supplement: Supplementary file 3 — Supplementary figure 3 [file 41419_2022_4575_MOESM3_ESM.png]

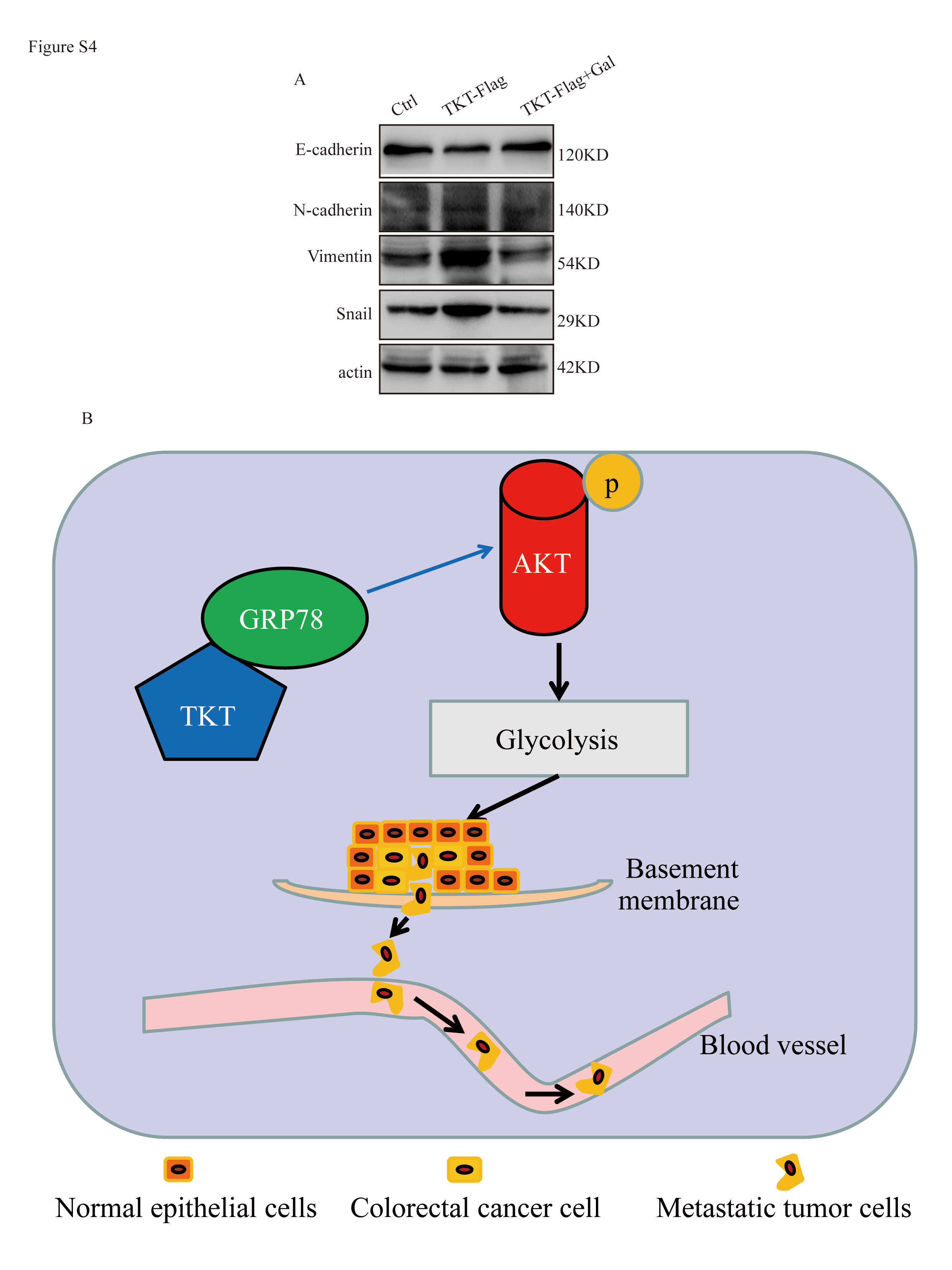

Supplement: Supplementary file 4 — Supplementary figure 4 [file 41419_2022_4575_MOESM4_ESM.png]
