# Supplementary material for: Transketolase promotes colorectal cancer metastasis through regulating AKT phosphorylation
Source: Cell Death Dis. 2022 Feb 2;13(2):99. doi: 10.1038/s41419-022-04575-5 (PMC8810869; doi:10.1038/s41419-022-04575-5)
Supplement: Supplementary file 5 — Supplementary figure legends [file 41419_2022_4575_MOESM5_ESM.doc]

**Figure S1:** The expression level of TKT was detected by Western blot analysis in six CRC cell lines (HT-29, DLD1, LOVO, SW620, HCT116, SW480) and normal colorectal epithelial cell line FHC.

**Figure S2:** Detecting the motility of HCT116 with TKT overexpression and knockdown by RTCA. *P < 0.05, **P < 0.01, ***P < 0.001.

**Figure S3:** TKT in CRC cells positively regulated tube formation. The numbers of complete tubular structures formed by HUVECs were counted for ±TKT OE/KD in SW480 and HCT116 cells.

**Figure S4: (A)** Protein expression levels of EMT markers in HCT116 cells. (B) The working model.
